# The Modern Tendency of Disease

**Published:** 1887-09

**Authors:** 


### The Modern Tendency of Disease.

Last month Dr. Milner Fothergill delivered three lectures on this topic, at his house in London. The gist of them was as follows:

"We have, of recent years, been studying disease, rather than the individual and the forces, internal and external, operating upon him. Of old a stout physique was the great matter for work and fighting, while life was country life.. Now the battle of life is fought with the brain, and life is no longer rural but urban. The strain upon the nervous system lends to visceral derangement. The digestion is impaired. Meat is largely eaten by town-dwellers because it is easily digested in the stomach. The consequence is, the liver is over taxed and reverts to the uric acid formation. This leads to changes in the vascular system and the kidneys, known as 'chronic Bright's disease,' for which he proposes to substitute the term 'vasorenal change.' The effects of brain-toil in the production of diseases connected with hepatic inadequacy, is seen in the prevalence at the present time, of diabetes and Bright's disease. Notably are these maladies common among Jews, the inhabitants of the United States of America, and of Bengal: all hard brain-workers. The demands upon the nervous system in school life, and the brain work of after life tell injuriously upon the hysique; while of girls, puberty is often imperfect, and marriage entails the aid of the gynecologist. Hard work is the order of the day with town-dwellers. 'The man who can get the most out of himself, is the man who can outstrip his competitors and get on in life,' he pithily remarked. And to do this the overworked man resorts to nervine tonics; and lastly to narcotics, when sleep fails him.

"Our altered habits of life required alterations in our food customs. The town-dweller cannot digest imperfectly cooked pound cakes and pastry, which give him the stomach-ache. He requires something which is easy of digestion, and therefore his dietary ought to consist largely of soluble carbohydrates. The imperfection of the digestive organs is very manifest in acute disease; and overworked men were apt to succumb to acute disease. Food almost independent of the digestive ferments was required to sustain the powers and prevent collapse. After the acute storm has blown over, much nervous prostration is left behind, entailing a slow and lengthened convalescence before the nervous system recovers its fitness for sustained work. The altered conditions of modern life were such as to require considerable modifications of food customs on health; while in disease not only had a change come over the treatment from an antiphlogistic to a sustaining regimen, but the necessity for material which could supply grape-sugar to the blood without requiring an action of the digestive ferments, was being widely recognized."

It is needless to add that he had appreciative audiences.

